# A Water Extract from *Chlorella sorokiniana* Cell Walls Stimulates Growth of Bone Marrow Cells and Splenocytes

**DOI:** 10.3390/nu14142901

**Published:** 2022-07-15

**Authors:** Susumu Ishiguro, Mary Roth, Ruth Welti, Mayme Loyd, Ravindra Thakkar, Morgan Phillips, Nicole Robben, Deepa Upreti, Ayaka Nakashima, Kengo Suzuki, Jeffrey Comer, Masaaki Tamura

**Affiliations:** 1 Department of Anatomy & Physiology, Kansas State University College of Veterinary Medicine, Manhattan, KS 66506, USA; isusumu@vet.k-state.edu (S.I.); loydmayme@gmail.com (M.L.); ravithakkar@vet.k-state.edu (R.T.); mphillips30@ksu.edu (M.P.); ndrobben@vet.k-state.edu (N.R.); deepa07@vet.k-state.edu (D.U.); jeffcomer@ksu.edu (J.C.); 2 Division of Biology, Kansas Lipidomics Research Center, Kansas State University, Manhattan, KS 66506, USA; mrroth@ksu.edu (M.R.); welti@ksu.edu (R.W.); 3 Euglena Co., Ltd., Minato-ku, Tokyo 108-0014, Japan; nakashima@euglena.jp (A.N.); suzuki@euglena.jp (K.S.)

**Keywords:** lipopolysaccharide-like substance, *Chlorella sorokiniana* cell wall extract, growth stimulation, bone marrow cells, splenocytes

## Abstract

A water extract derived from the isolated cell walls of *Chlorella sorokiniana* (*C*. *sorokiniana*, Chlorella water extract, CWE) was analyzed for the presence of lipopolysaccharide (LPS)-related material via the Limulus amebocyte lysate (LAL) assay and evaluated for its growth stimulation effect on the bone marrow cells and splenocytes in vitro cell cultures. The extract contained low levels of LPS-related material, and a mass spectrum suggested that the extract contained many components, including a low level of a lipid A precursor, a compound known as lipid X, which is known to elicit a positive response in the LAL assay. Treatment with the CWE dose- and time-dependently stimulated the growth of mouse bone marrow cells (BMCs) and splenocytes (SPLs). Treatment with the CWE also increased specific BMC subpopulations, including antigen-presenting cells (CD19^+^ B cells, 33D1^+^ dendritic cells and CD68^+^ macrophages), and CD4^+^ and CD8^+^ T cells, but decreased the number of LY6G^+^ granulocytes. Treatment with the CWE also increased cytokine mRNA associated with T cell activation, including TNFα, IFNγ, and granzyme B in human lymphoblasts. The present study indicates that the cell wall fraction of *C.* *sorokiniana* contains an LPS-like material and suggests a candidate source for the bioactivity that stimulates growth of both innate and adaptive immune cells.

## 1. Introduction

*Chlorella* is a genus of unicellular green algae found in fresh water and seawater [1]. Because this alga contains a variety of nutrients such as amino acids, carbohydrates, vitamins, minerals, and dietary fiber, as well as bioactive components such as pigments, organic compounds, fatty acids, peptides etc., it is taken as a nutritional and functional dietary supplement throughout the world [2,3,4,5]. Enhancement of specific nutritional/functional properties can be attainable by managements of culture conditions of algae [6,7,8,9]. In addition, preclinical studies have shown that whole dried powder and/or water/organic solvent extracts of *Chlorella* have therapeutic effects against various cancers [10,11,12,13,14,15,16,17]. These effects are attributable to the stimulation of host anti-tumor immune responses [11,14,16]. However, the origin and nature of the bioactive component(s) is yet to be fully clarified.

Lipopolysaccharide (LPS) is a glycolipid found in the bacterial outer membrane of Gram-negative bacteria [18,19], which is recognized as endotoxin by innate immune systems via Toll-like receptor 4 (TLR4) and induce inflammatory responses [20]. LPS is composed of three parts: O-antigen, Core oligosaccharide, and lipid A; O-antigen structures are highly diverse, while the lipid A structure is conserved at the species level [18]. It was reported that LPS-like molecules are found in some organisms such as Gram-negative photosynthetic prokaryotes, cyanobacteria (also called as blue-green alga) [21]; promastigotes, *Leishmania* [22]; algae, *Prototheca* [23]; and land plants, *Arabidopsis thaliana* [24]. In addition, LPS from Gram-negative photosynthetic prokaryotes, *Rhodobacter*, acts as LPS antagonist which shows anti-oxidant and anti-inflammatory effects [25] as well as anti-cancer effects prevent lung metastasis [26]. High doses of LPS lead to excessive immune responses, called endotoxin shock, which is a cause of sepsis [27]. However, low doses of LPS stimulate functional activation and maturation of immune cells, therefore, it is used as an adjuvant of vaccines; monophosphoryl lipid A as an adjuvant for cervical cancer vaccines, also known as human papillomavirus (HPV) vaccines [28].

The Chlorella cell wall is a thick membrane composed of a large amount of insoluble polysaccharide, a relatively small amount of protein/glycoprotein, and unidentified materials [29,30]. It is also reported that the cell wall contains lipopolysaccharide-like immune-reactive substances, although their chemical structures have yet to be determined [31]. Since the Chlorella cell wall is unique in structure and composition and makes up a relatively large portion of the Chlorella body, it is of interest to study the immune response related activities of the water extract from the Chlorella cell wall.

Our recent study suggested that water extract of the cell wall fraction from *Chlorella sorokiniana* attenuated colon carcinoma growth in cell culture and mice by inducing apoptosis of cancer cells [32]. This extract also stimulated cytotoxicity of T cells in three-dimensional spheroid culture with colon carcinoma cells, however a bioactive component(s) in the extract is yet to be identified. Here, we report for the first time that water extract from the cell wall fraction of *Chlorella sorokiniana* (CWE), which stimulates the growth of bone marrow cells and splenocytes in vitro in cell culture, contains a low level of lipopolysaccharide (LPS)-like substance (LPS-LS) which includes a molecule similar to the lipid X found in *Arabidopsis thaliana* [24].

## 2. Materials and Methods

### 2.1. Preparation of CWE

Water extract from the *Chlorella sorokiniana* cell wall fraction (CWE) was initially prepared by a proprietary method developed by the Euglena Co. Ltd. (Minato-ku, Tokyo, Japan). Briefly, *Chlorella sorokiniana* collected from Ishigaki, Japan, was aseptically cultured in modified Hannan and Patouille medium [33] supplemented with glucose as a source of carbon. The cell walls spontaneously suspended in Chlorella culture media were separated from intact cell bodies of *Chlorella sorokiniana* by nozzle separator (Y-250H: Saito Separator Ltd., Ohta-ku, Tokyo, Japan). The resultant Chlorella cell wall fraction was washed with deionized water twice by centrifuging at 6800× *g* for 5 min and lyophilized.

To extract bioactive components from the Chlorella cell wall fraction, lyophilized cell walls were suspended in phosphate buffered saline (PBS) at a concentration of 40 mg/mL and incubated at 4 °C for 12 h and then at 37 °C for 30 min with periodic sonication for 30 s and vortex mixing. Insoluble materials composed of Chlorella cell walls were removed by two steps of centrifugation at 2300× *g* and 11,800× *g*, respectively, at room temperature for 10 min each. The resultant supernatant fraction was filtered through a 0.22 µm disk filter (Midwest Scientific, Valley Park, MO, USA) and stored at −20 °C until use. This fraction was designated the CWE partially purified from Chlorella cell walls and subjected to the experiments described below. The amount of LPS-LS in this preparation was determined by the Limulus amebocyte lysate (LAL) assay (Pierce Chromogenic Endotoxin Quantitation Kit, Thermo Fisher Scientific, Waltham, MA, USA).

### 2.2. Analysis of Chlorella CWE for Lipid A and Lipid X by Mass Spectrometry

Chlorella cell walls were subjected to the method described by Henderson et al. for the preparation of Lipid A [34]. Lipid A from *Escherichia coli* (*E*. *coli*) was purchased from Sigma–Aldrich (St. Louis, MO, USA). Lipids were extracted from *Arabidopsis thaliana* (*A*. *thaliana*) was extracted by the method described by Welti et al. [35] and further extracted using “solvent H” as carried out by Markham et al. [36], based on Toledo et al. [37]. All samples were dissolved in chloroform/methanol/300 mM ammonium acetate in water (35/66.5/3.5, *v*/*v*/*v*), ionized by electrospray and subject to fragmentation in a Waters Xevo TQ-S triple quadrupole mass spectrometer (Waters Corporation, Milford, MA, USA) by direct infusion.

### 2.3. Electron Microscopy

For both scanning electron microscopy (SEM) and transmission electron microscopy (TEM), the lyophilized Chlorella cell wall fraction was rehydrated with PBS, fixed with Trump’s fixative (1% glutaraldehyde and 4% formaldehyde in a 0.1 M phosphate buffer at pH 7.4) overnight at 4 °C, post-fixed with 1% osmium tetroxide in a 0.2 M phosphate buffer for 1 h, and dehydrated with a series of graded ethanol solutions. For the SEM analysis, ethanol in the dehydrated cell wall sample solution was replaced with hexamethyldisilazane by centrifugation and the membrane sample was sputtered with palladium using Denton Vacuum Desk II sputter coater (Denton Vacuum, Moorestown, NJ, USA). Sputter-coated Chlorella cell walls were analyzed using Hitachi S-3500 N Scanning Electron Microscope (Hitachi Science Systems Ltd., Minato-ku, Tokyo, Japan) at an accelerating voltage of 10 kV. For the TEM investigation, the Chlorella cell walls dehydrated with ethanol were washed with acetone and embedded in Spurr resin, followed by polymerization of sample block in flat embedding molds. The sample was thin-sectioned at a thickness of 700–900 Å using a Leica UC7 ultramicrotome (Leica Biosystems Inc., Buffalo Grove, IL, USA) and placed on a 200-mesh copper TEM grid. Ultrathin sections were analyzed using a FEI Tecnai G2 Spirit BioTWIN transmission electron microscope (FEI Company, Hillsboro, OR, USA) at an accelerating voltage of 80 kV. Electron micrographs were taken with a Tecnai 12 (FEI Company, Hillsboro, OR, USA) microscope, equipped with a Gatan CCD camera (Gatan, Inc., Pleasanton, CA, USA).

### 2.4. Cell Culture

The Jurkat human lymphoblast cell line (TIB-152) was purchased from American Type Culture Collection (ATCC; Manassas, VA, USA). The Jurkat cells were cultured in RPMI 1640 (Mediatech, Inc., Manassas, VA, USA) supplemented with 10% *v*/*v* FBS (EQUITECH-BIO Inc.; Kerrville, TX, USA) and 1% *v*/*v* penicillin-streptomycin (Lonza Rockland, Inc.; Allendale, NJ, USA). Cell culture was carried out at 37 °C in a humidified air atmosphere containing 5% CO_2_. The cell line was authenticated by short tandem repeat (STR) DNA profiling. Both the cells were maintained in low passages (<15) for this study.

### 2.5. Animals

Female Balb/c mice were obtained from Charles River Laboratories International, Inc. All mice were housed in a clean facility under controlled conditions of temperature (20–26 °C), with 30–70% relative humidity and light (12:12 h light–dark cycles) and acclimatized for 10 days. All mice were housed humanely according to university, state, and federal guidelines (AAALAC) in the AAALAC-accredited animal resource facilities of the Kansas State University College of Veterinary Medicine. The mice’s condition was observed daily, and body weights were obtained every other day. All animal experiments adhered strictly to protocols approved by the Kansas State University Institutional Animal Care and Use Committee (Protocol # 4346) and Institutional Biosafety Committee (Registration # 1317).

### 2.6. Effect of CWE on the Growth of Bone Marrow Cells and Splenocytes in Cell Culture

The effect of CWE on the proliferation of bone marrow cells (BMCs) and splenocytes (SPLs) was evaluated by MTT (3-(4,5-dimethylthiazol-2-yl)-2,5-diphenyltetrazolium bromide) assay in a 96-well plate. The Balb/c mice were sacrificed by exposure to saturated CO_2_ followed by cervical dislocation. BMCs were harvested from the bone marrow in the hind legs and cultured in RPMI 1640 supplemented with 10% *v*/*v* FBS, 1% *v*/*v* penicillin-streptomycin. SPLs were collected from the spleens and cultured in RPMI 1640 supplemented with 10% *v*/*v* FBS, 1% *v*/*v* penicillin-streptomycin, and 20 µM 2-mercaptoethanol. BMCs (2 × 10^5^ cells/well) and SPLs (5 × 10^5^ cells/well) were cultured in a 96-well plate. Cell proliferation was evaluated for its dose- (at 0.1, 1 and 10 µg/mL CWE) and time-dependencies (at 24, 48, 72 and 96 h of treatment with 0.1, 1 and 10 µg/mL CWE). PBS and 100 ng/mL authentic LPS (*Escherichia coli* 026:B6; Sigma–Aldrich, St. Louis, MO, USA) served as negative and positive controls, respectively.

### 2.7. Flow Cytometry Analysis of CWE-Induced Proliferation of BMCs In Vitro

BMCs (5 × 10^6^ cells) were seeded into a 6-well plate and incubated with 10 µg/mL CWE for 48 h. Cells were immunostained with anti-CD4 (H129.19; helper T cells), anti-CD8b (YTS156.7.7; cytotoxic T cells), anti-CD19 (6D5; B cells), anti-DC marker (33D1; dendritic cells), anti-LY6G (1A8; neutrophil) and anti-CD68 (FA-11; macrophage) antibodies. Antibodies which match the host species and the class with primary antibodies above were used for the isotype control. All antibodies were obtained from BioLegend (San Diego, CA, USA). The changes of cell populations were analyzed by flow cytometry (BD LSRFortessa X-20; BD Biosciences, San Jose, CA, USA) and analyzed by BD FACSDiva (BD Bioscience, San Jose, CA, USA). PBS and authentic LPS (100 ng/mL) served as negative and positive treatment controls, respectively.

### 2.8. Effect of CWE on the Growth of Jurkat Cells

Jurkat cells (1000 cells/well) were seeded in a 96-well plate and treated with CWE (1–100 µg/mL) and authentic LPS (0.1–10 µg/mL) after 24 h. Cell proliferation was evaluated by MTT assay at 48 h after the treatment.

### 2.9. Cytokine Expression in CWE-Treated Jurkat Cells

Gene expression of tumor necrosis factor alpha (TNFα), interferon gamma (IFNγ) and granzyme B (GZMB) in CWE-treated Jurkat cells were measured by reverse transcription quantitative polymerase chain reaction (RT-qPCR). Jurkat cells (1 × 10^5^ cells/well) were seeded into a 12-well plate. After 24 h, the cells were treated with 1 or 10 µg/mL of CWE, or 5, 10 or 100 ng/mL authentic LPS. Total RNA was purified at 24 and 48 h after treatment, using the reagent TRIzol (InvitroGen; Thermo Fisher Scientific, Waltham, MA, USA). One step RT-qPCR was performed using the iTaq Universal SYBR Green One-Step Kit (Bio-Rad; Hercules, CA, USA). The RT-qPCR was performed as follows: 45 cycles of 15 s at 95 °C, and 60 s at 60 °C. The results were quantified by the comparative Ct method [38]. Table 1 displays the sequences of the primers.

### 2.10. Morphological Observation of CWE-Treated Jurkat Cells

To evaluate the morphological differentiation of immature T lymphoblasts, Jurkat cells were cultured three-dimensionally (3D) as described previously with slight modifications [39]. The cells were treated with CWE (10 µg/mL), or authentic LPS (10 ng/mL) on Day 1 (24 h after Jurkat cell seeding) and Day 4 (96 h after Jurkat cell seeding). The image of Jurkat cells was taken by an inverted microscope IX51 (Olympus America Inc., Center Valley, PA, USA) equipped with cellSens Dimension software (Olympus America Inc., Center Valley, PA, USA) at Day 7.

### 2.11. Statistical Analysis

All values are expressed as the mean ± standard deviation of mean. For all in vitro experiments, statistical significance was assessed by an unpaired t-test or ANOVA followed by Tukey’s test. All experiments were conducted with multiple sample determinations with several samples (*n* = 3–5). Statistical significance was set at *, *p* < 0.05.

## 3. Results

### 3.1. Morphological Analysis Revealed the Chlorella Cell Wall Fraction Is Composed of Only Cell Walls and Membranes

**Figure 1:**
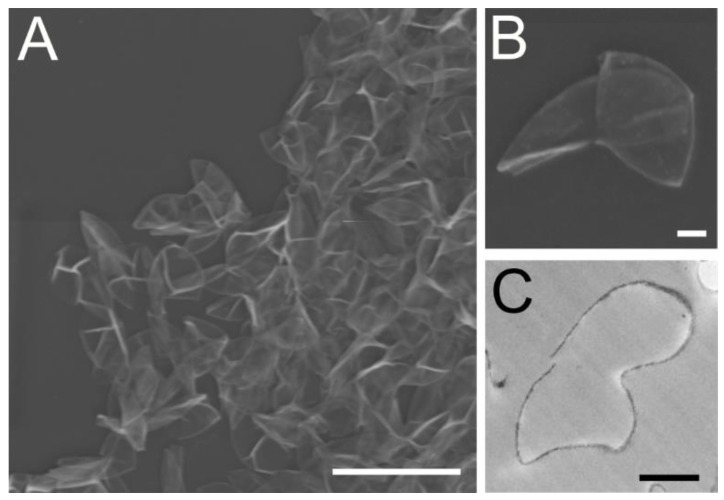
Scanning panels (**A**,**B**) and transmission panel (**C**) electron micrographs of washed Chlorella cell wall fraction. Scale bars in the panels (**A**–**C**) represent 10 µm, 1 µm, and 1 µm, respectively.

The morphological analyses by both SEM and TEM clearly indicate that the washed cell wall fraction is composed of only Chlorella cell walls and membranes and no intact Chlorella cell bodies or bacteria contaminated this fraction (Figure 1).

To clarify whether the bioactivity is attributable to an LPS-like substance in the final extract of the Chlorella cell wall fraction, the “LPS” levels in the final extract were determined using a commercially available Limulus amebocyte lysate (LAL) assay. This assay reacts positively with LPS and related compounds, including the LPS core lipid, lipid A, and its precursor, lipid X [40,41]. The results of the assay demonstrated that the final extract contained low levels of LPS-like material (average 0.200 ± 0.002 ng per 1 µg dry weight of preparations, *n* = 3).

### 3.2. Mass Spectrometry Suggests the Presence of Lipid X, but Not Lipid A, in Chlorella Cell Wall Fraction

**Figure 2:**
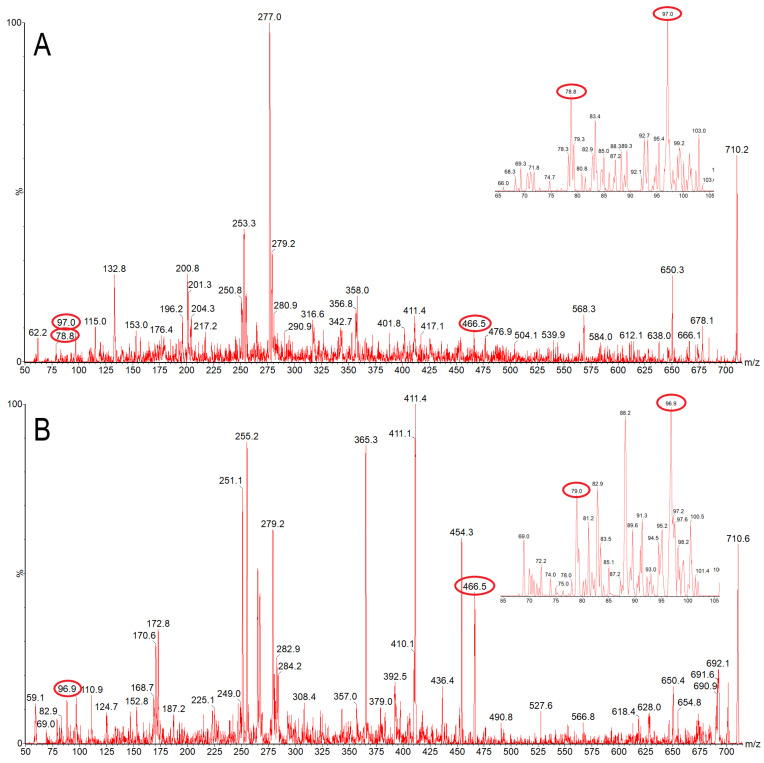
Product ion spectra of *m/z* 710.4, the *m/z* of the [M-H]^−^ ion of lipid X. (**A**) *Arabidopsis thaliana* extract, (**B**) Chlorella cell wall fraction. Insets show the *m/z* range from 65 to 105. Fragments characteristic of lipid X are circled. Characteristic fragments include *m/z* 79, 97, 240, and 466, of which *m/z* 79, 97, and 466 were detected in both species.

Fragmentation of the [M-H]^−^ ion of commercially obtained *E*. *coli* lipid A (*m/z* 1796) in negative ion mode generated a spectrum with large pyrophosphate (*m/z* 177 and 159) and phosphate (*m/z* 97 and 79) peaks, as observed by Jones et al. [42]. Those fragments were not observed as products of *m/z* 1796 in the Chlorella cell wall extract. The plant *A*. *thaliana*, which contains genes homologous to those in the *E*. *coli* lipid A biosynthetic pathway, does not contain observable lipid A, but does contain lipid X, 2,3-diacylglucosamine-1-phosphate, an intermediate in lipid A biosynthesis [24]. Thus, we considered whether the Chlorella cell wall might contain lipid X. Li et al. demonstrated that there are four characteristic fragments of the lipid X [M-H]^−^ ion (*m/z* 710): phosphate (*m/z* 97 and 79), glucosamine-phosphate (*m/z* 240), and hydroxymyristoylglucosamine-phosphate (*m/z* 466) [24]. Negative-ion fragmentation of *m/z* 710 in both a wild-type *A*. *thaliana* extract and the Chlorella cell wall extract generated peaks at *m/z* 466, *m/z* 97, and *m/z* 79, among others that were likely derived from other compounds of the same nominal mass (Figure 2). These results suggest that a small amount of lipid X may be present in the Chlorella cell wall.

### 3.3. CWE Treatment Stimulated the Growth of Bone Marrow Cells and Splenocytes

**Figure 3:**
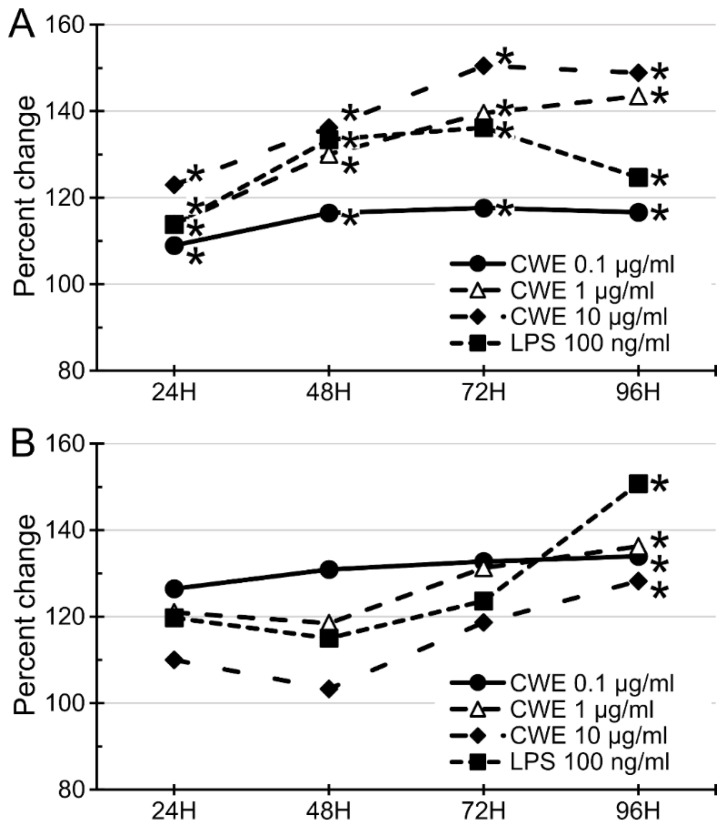
CWE dose- and time-dependently stimulated the growth of murine bone marrow cells (BMCs) and splenocytes (SPLs) in cell culture. CWE treatment dose-dependently (0.1–10 µg/mL) and time-dependently (24, 48 72 and 96 h) stimulated the growth of mouse BMCs (**A**) but not SPLs (**B**), 100 ng/mL LPS served as a positive control. The cell growth was evaluated by MTT (3-(4,5-dimethylthiazol-2-yl)-2,5-diphenyltetrazolium bromide) assay. The data was shown as percent change compared with phosphate buffered saline (PBS)-treated control. *, *p* < 0.05 compared to PBS-treated control (*n* = 3).

CWE treatment (0.1–10 µg/mL) significantly increased the growth of BMCs in a dose- and time-dependent manner (Figure 3A). However, the same dosage range of CWE treatment showed a highest growth stimulation of SPL at the lower dosage (0.1 µg/mL), but the higher dosages (1 and 10 µg/mL) showed less growth stimulation than that by the lower dosage (Figure 3B). The positive control, LPS derived from *Escherichia coli* 026:B6, also exhibited similar patterns with the CWE on BMCs. Comparison of CWE and authentic LPS demonstrated that their growth stimulation patterns are very similar. Hence, the aqueous extract of the Chlorella cell wall fraction contains LPS-like bioactivity, justifying our designation of this substance as an LPS-like substance. However, the functionality of the CWE is apparently different from that of the bacterial LPS; CWE can efficiently stimulate growth of hematopoietic precursor cells in bone marrow, but its effect on differentiated lymphocytes in spleen is limited, whereas the bacterial LPS can stimulate growth of hematopoietic precursor cells in bone marrow and differentiated lymphocytes in the spleen.

### 3.4. CWE Treatment Increased Lymphocyte and Antigen-Presenting Cell Populations but Decreased Neutrophil Population in Bone Marrow Cells

**Figure 4:**
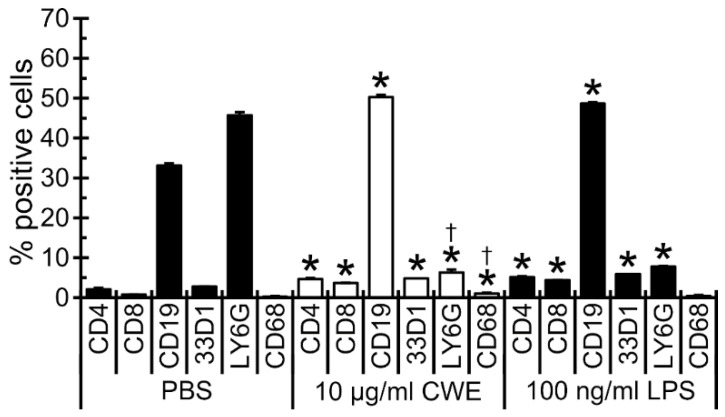
CWE treatment increased various immune cell populations in primary cultured mouse bone marrow cells (BMCs). Percentage of the population of BMCs treated with 10 µg/mL CWE and 100 ng/mL LPS for 48 h were evaluated by flow cytometry. These BMCs were labeled with various antibodies and relative quantities were analyzed by flow cytometry. PBS and LPS served as negative and positive controls, respectively. *, *p* < 0.05 compared to PBS control group; †, *p* < 0.05 compared to LPS-treated group (*n* = 3).

CWE treatment stimulated the growth of BMCs as a whole (Figure 3A). Detailed changes of immune cell populations in BMCs were analyzed by flow cytometry. As shown in Figure 4, treatment with 10 µg/mL CWE increased CD4^+^ (56.1% increase vs. the PBS control group, *p* < 0.05), CD8^+^ (78.2% increase, *p* < 0.05), CD19^+^ (34.2% increase, *p* < 0.05), CD68^+^ (77.6% increase, *p* < 0.05) and 33D1^+^ (41.4% increase, *p* < 0.05) cells, whereas the LY6G+ cell population was significantly decreased (86.2% decrease, *p* < 0.05) as compared with the PBS control group. Treatment with LPS (100 ng/mL) elicited a similar pattern of cell growth as that with CWE except CD68^+^ macrophage population (LPS did not change this cell population, Figure 4). These results suggest that increases in cell populations caused by CWE treatment are primarily related to the adaptive immune system.

### 3.5. CWE Treatment Induced Expression of T Lymphocyte Activation-Associated Cytokines and Caused Morphological Differentiation of Lymphoblasts

**Figure 5:**
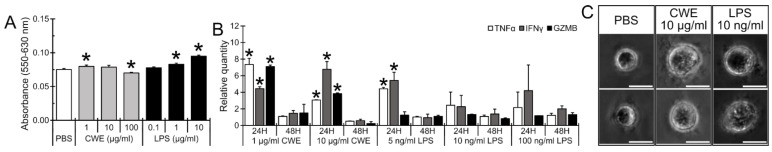
CWE treatment increased cytokine expressions and induced morphological differentiation in Jurkat cells. (**A**) Cell proliferation of Jurkat cells was evaluated by MTT assay in the presence or absence of CWE (1–100 µg/mL) or LPS (0.1–10 µg/mL) at 48 h after the treatment (*n* = 3). (**B**) T cell activation-associated cytokine expression in Jurkat cells treated with 1 or 10 µg/mL CWE, or 5, 10, or 100 ng/mL LPS at 24 and 48 h after treatment was measured by RT-qPCR. *, *p* < 0.05 compared to PBS control group (*n* = 4). (**C**) The Jurkat cells were grown in a U-shaped agar matrix (Day 0). The CWE (10 µg/mL) or LPS (10 ng/mL) was treated twice at Day 1 and Day 4. Typical pictures of CWE- or LPS-treated Jurkat cells in the agar matrix at Day 7. Scale bar, 20 µm.

As shown in Figure 4, CWE treatment increased the T lymphocyte population. In order to evaluate the effect of CWE for functional activation of T lymphocytes, the effect of CWE on cell proliferation and gene expression of TNFα, IFNγ, and granzyme B (GZMB) in Jurkat cells was measured by MTT assay and real-time PCR, respectively. Although CWE (1–100 µg/mL) only slightly altered the cell proliferation (a low concentration slightly increased, but a high concentration slightly decreased cell proliferation Figure 5A), LPS from *E*. *coli* significantly stimulated the cell proliferation in a dose-dependent manner. As shown in Figure 5B, the expression of TNFα, IFNγ, and GZMB was significantly upregulated at 24 h after CWE treatment and returned to same level as that of the PBS control group at 48 h. In contrast, LPS increased only TNFα and IFNγ expression, but not GZMB expression. In addition, when lymphoblasts were treated with CWE, they were enlarged and exhibited significant morphological differentiation (Figure 5C). The same kind of morphological differentiation was also observed in LPS-treated Jurkat cells (Figure 5C). These results suggest that CWE treatment stimulates activation of T lymphocytes. It also suggests that stimulatory activity of CWE for lymphoblast is different from that by an authentic LPS.

## 4. Discussion

Chlorella has been used as a dietary supplement with high nutritional value worldwide, and a large number of publications indicate that Chlorella extracts may serve as sources of therapeutics against various diseases including cancers [14,43,44,45,46]. Practically all these studies have used a water or alcohol extract from whole Chlorella including crushed Chlorella powders. However, since whole Chlorella contains a large number of potentially bioactive substances including cytotoxic materials, it seems likely that most reported medicinal activities are due to a mixture of bioactive substances. On the other hand, specific roles for certain bioactive substances from Chlorella have been demonstrated. The polysaccharide of *C*. *pyrenoidosa* and *C*. *sorokiniana* stimulates innate immunity via TLR4 [47,48]. It has also been reported that a glycoprotein in *C*. *vulgaris* stimulates cytokine production in adherent splenocytes through the TLR2 signaling pathway in mice, suggesting that this glycoprotein stimulates innate immunity via TLR2 [49]. In addition, it should be noted that the cell wall of Chlorella contains lipopolysaccharide-like immunoreactivity [31,50]. Although it is not easy to pinpoint a single bioactive compound in natural products, it can be useful to determine the subcellular components from which the bioactive substances originate and their general chemical makeup. In an effort to identify the origin of the bioactivity and to reduce the number of bioactive substances in the extract, the present study focused on the cell wall fraction of *Chlorella sorokiniana* and its water extract, and on evaluating its immune modulatory abilities in in vitro.

Authenticity of the Chlorella cell wall was examined using both scanning and transmission electron microscopies. These morphological analyses clearly indicated that the materials used for the present study were composed entirely of cell walls and plasma membranes derived from Chlorella, and no evidence of intact cell body or bacterial contamination was found (Figure 1). The morphologies of the cell walls and associated membranes were identical to those of previously published paper by Northcote et al. [30].

Potential contamination by bacterial LPS was excluded by lack of detectable lipid A by mass spectrometry. On the other hand, the LAL assay, which responds to both lipid A, which is found in Gram-negative bacteria, but not in plants, and lipid X, which is found in both bacteria and plants, was present at low, but measurable levels (0.2 ng/µg dry extract). Mass spectrometry provided suggestive evidence for a low-level presence of the lipid A precursor, lipid X, but not lipid A, in the Chlorella cell wall fraction. As shown in Figure 2, in a parallel analysis of the plant *A*. *thaliana*, only lipid X was detected, while lipid A was not observed, as reported by Li et al. [24]. These data suggest that lipid X is potentially present in the Chlorella cell wall extract. However, whether lipid X is primarily responsive for CWE-dependent bioactivities awaits further studies, including complete purification and identification of its chemical nature.

The effect of CWE on the growth of murine bone marrow cells and splenocytes was evaluated in cell culture-based studies. Treatment with the CWE significantly increased the growth of immune cells in the bone marrow and spleen in a time- and dose-dependent manner (Figure 3). Clarification of the specific cell populations influenced by this treatment revealed that the CWE treatment increased the numbers of lymphocytes (CD4^+^ and CD8^+^ T cells, and CD19^+^ B cells) and antigen-presenting cells (33D1^+^ dendritic cells and CD68^+^ macrophages) (Figure 4).

Furthermore, gene expression of T lymphocyte activation-associated cytokines was also found to be upregulated by CWE treatment in Jurkat cells (Figure 5B). CWE treatment also induced morphological differentiation in Jurkat cells in 3D culture (Figure 5C). However, gene expression profiles of T cell activation-associated cytokines in bacterial LPS-treated Jurkat cells were slightly different from those by CWE (Figure 5B). Although Jurkat cells are human lymphoblasts derived from an acute T cell leukemia patient, they have been used as a model cell for normal T lymphocytes. For example, Jurkat cells were used for the evaluation of IL-2-dependent granzyme B production, which is a marker of T lymphocyte activation [51,52]. Taken together, these results imply that CWE treatment appears to induce functional differentiation in T lymphocytes. Therefore, it appears that CWE is capable of T lymphocyte activation as well as leukocyte growth stimulation, which is very similar to bacteria cell wall-derived LPS [53,54]. In this regard, it should be noted that the cell wall of Chlorella contains lipopolysaccharide-like immunoreactivity [31,50]. In the present study, however, CWE slightly, but clearly, inhibited growth of Jurkat cells, whereas authentic LPS stimulated their growth in a parallel experiment (Figure 5A). Furthermore, analysis of BMC responses to CWE or LPS showed that CD68^+^ macrophages respond only to CWE treatment (Figure 4). It is apparent that the action of CWE is different from LPS in stimulation of Jurkat and macrophages. These results suggest that the bioactive compound(s) in the Chlorella cell wall fraction is apparently distinct from bacterial LPS. However, determination of the detailed chemical nature of such bioactive compounds in the Chlorella cell wall fraction awaits future study. On the other hand, this CWE-induced direct differentiation and/or activation of T lymphocytes suggests that CWE is potentially a useful agent for cancer immune therapy, applicable to both primary and metastatic cancer. To the best of our knowledge, this is the first study to report that the extract from the Chlorella cell wall fraction stimulates the growth of immune cells in the spleen and bone marrow and induces the functional differentiation of T lymphocytes.

## 5. Conclusions

Analysis of CWE from the cell wall fraction of *Chlorella sorokiniana* indicated the presence of LPS-like immunoreactivity, and mass spectrometry suggested that a small amount of lipid X may be present in the Chlorella cell wall. The CWE significantly stimulates the growth of primary cultured mouse BMCs and SPLs. Treatment with the CWE also significantly increased the number of lymphocytes (CD4^+^ and CD8^+^ T cells, and CD19^+^ B cells) and antigen-presenting cells (33D1^+^ dendritic cells and CD68^+^ macrophages) in primary cultured mouse BMCs. In a 3D culture, the CWE treatment caused morphological differentiation of lymphoblasts, i.e., Jurkat cells. These data show that CWE could be a useful agent for the stimulation of anti-tumor or anti-microbial immunity.

## Figures and Tables

**Table 1:** Primers used for RT-qPCR.

Primer		Sequence	Size
Human	Forward (5′-3′)	GCCAGAATGCTGCAGGACTT	63 bp
TNFα	Reverse (5′-3′)	GGCCTAAGGTCCACTTGTGTCA	
Human	Forward (5′-3′)	AGGGAAGCGAAAAAGGAGTCA	64 bp
IFNγ	Reverse (5′-3′)	GGACAACCATTACTGGGATGCT	
Human	Forward (5′-3′)	ATGAGACAGCAACCATTGTAGAATTT	87 bp
IL-2	Reverse (5′-3′)	CACTTAATTATCAAGTCAGTGTTGAGATGA	
Human	Forward (5′-3′)	TGCAGGAAGATCGAAAGTGCG	180 bp
GZMB	Reverse (5′-3′)	GAGGCATGCCATTGTTTCGTC	
18S	Forward (5′-3′)	GAGGTTCGAAGACGATCAGA	315 bp
	Reverse (5′-3′)	TCGCTCCACCAACTAAGAAC	

## Data Availability

All data is available upon request to the corresponding author.
